# Acetylglutamine facilitates motor recovery and alleviates neuropathic pain after brachial plexus root avulsion in rats

**DOI:** 10.1186/s12967-023-04399-7

**Published:** 2023-08-23

**Authors:** Lin Wu, Shuangxi Chen, Bing He, Guijuan Zhou, Yan Xu, Guanghua Zhu, Juan Xie, Limin Deng, Xuanwei Wen, Sijing Li, Zijian Xiao

**Affiliations:** 1 Department of Neurology, Multi-Omics Research Center for Brain Disorders, The First Affiliated Hospital, Hengyang Medical School, University of South China, Hunan 421001 Hengyang, China; 2 Clinical Research Center for Immune-Related Encephalopathy of Hunan Province (The First Affiliated Hospital), Hengyang Medical School, University of South China, Hunan 421001 Hengyang, China; 3 Department of Neurology, The Second Affiliated Hospital, Hengyang Medical School, University of South China, Hunan 421000 Hengyang, People’s Republic of China

**Keywords:** Acetylglutamine (NAG), Brachial plexus root avulsion (BPRA), Motoneuron (MN), Inflammation, Pyroptosis

## Abstract

**Background:**

Brachial plexus root avulsion (BPRA), a disabling peripheral nerve injury, induces substantial motoneuron death, motor axon degeneration and denervation of biceps muscles, leading to the loss of upper limb motor function. Acetylglutamine (N-acetyl-L-glutamine, NAG) has been proven to exert neuroprotective and anti-inflammatory effects on various disorders of the nervous system. Thus, the present study mainly focused on the influence of NAG on motor and sensory recovery after BPRA in rats and the underlying mechanisms.

**Methods:**

Male adult Sprague Dawley (SD) rats were subjected to BPRA and reimplantation surgery and subsequently treated with NAG or saline. Behavioral tests were conducted to evaluate motor function recovery and the mechanical pain threshold of the affected forelimb. The morphological appearance of the spinal cord, musculocutaneous nerve, and biceps brachii was assessed by histological staining. Quantitative real-time PCR (qRT‒PCR) was used to measure the mRNA levels of remyelination and regeneration indicators in myocutaneous nerves. The protein levels of inflammatory and pyroptotic indicators in the spinal cord anterior horn were measured using Western blotting.

**Results:**

NAG significantly accelerated the recovery of motor function in the injured forelimbs, enhanced motoneuronal survival in the anterior horn of the spinal cord, inhibited the expression of proinflammatory cytokines and pyroptosis pathway factors, facilitated axonal remyelination in the myocutaneous nerve and alleviated atrophy of the biceps brachii. Additionally, NAG attenuated neuropathic pain following BPRA.

**Conclusion:**

NAG promotes functional motor recovery and alleviates neuropathic pain by enhancing motoneuronal survival and axonal remyelination and inhibiting the pyroptosis pathway after BPRA in rats, laying the foundation for the use of NAG as a novel treatment for BPRA.

## Introduction

Over the years, the increase in the number of domestic motorcycles and the lack of safety awareness of drivers has been paralleled by an increase in traffic accidents, leading to a surge in the prevalence of brachial plexus root avulsion (BPRA) [1]. BPRA induces the massive death in motoneurons, the degeneration of motor axons and targeted biceps muscle denervation, eventually leading to the loss of motor function in the upper extremity [2, 3]. The permanent disability of the upper extremities caused by BPRA significantly affects patients and represents a burden for families and society [4]. To facilitate motor function recovery after BPRA, different types of treatments, such as surgical nerve reimplantation [5], can be performed. However, the axons of motor neurons grow too slowly to reinnervate target muscles, resulting in poor clinical prognosis after nerve reimplantation [6]. It has been shown that avulsion of ventral roots may result in extensive degeneration of motoneurons in the spinal cord anterior horn [7]. Koliatsos et al. demonstrated that ventral root avulsion caused 80% motor neuron retrograde death within the first 2 weeks [8]. Promoting the survival of injured motor neurons is a prerequisite for functional motor recovery after BPRA [9]. Accordingly, the exploration of novel medical approaches is essential to accelerate the survival of motoneurons combined with reimplantation surgery during BPRA treatment. Intermittent shooting pain induced by BPRA is commonly quite difficult to treat [10].

Acetylglutamine (N-acetyl-L-glutamine, NAG), which is derived from glutamine and generated by glutamine acetylation [11], can produce two metabolites: glutamic acid (Glu) and g-aminobutyric acid (GABA) [12]. GABA can promote acetylcholine activity and maintain normal brain function [13]. Moreover, Glu is an excitatory neurotransmitter that is tightly associated with the plasticity of neurons, playing a vital role in neuron growth and synapse generation [14]. Accumulating evidence has confirmed that NAG can play an essential neuroprotective role in cerebral ischemia‒reperfusion lesions via neuronal apoptosis and inflammation attenuation [13]. NAG has been reported to cross the blood‒brain barrier (BBB) and play multiple beneficial roles in neurological diseases [12]. Since they are easily accessible and affordable, NAG injections are extensively used to treat hemiplegia, hepatic coma, brain trauma, and cerebral apoplexy sequelae.

Given the essential effects of NAG on the normal and diseased nervous system, we hypothesized that NAG could promote motor and sensory function recovery in rats following BPRA. Herein, we showed that NAG could inhibit inflammation and promote motoneuron survival, axon remyelination, and biceps brachii atrophy to accelerate motor function recovery and alleviate neuropathic pain in rats following BPRA.

## Materials and methods

### Animals

Adult male Sprague Dawley (SD) rats (210–250 g) were purchased from Changsha Tianqin Biotechnology Co., Ltd. and housed in the Department of Laboratory Animal Science of the University of South China. The light cycle was maintained at 12 h light and 12 h dark, the room temperature (RT) was at 18–22 °C, and the humidity was controlled between 50 and 60%. The rats had free access to drinking water and food. All experimental animal procedures were approved by The Laboratory Animal Ethics Committee of The First Affiliated Hospital of the University of South China.

### Brachial plexus avulsion and reimplantation surgery

**Fig. 1:**
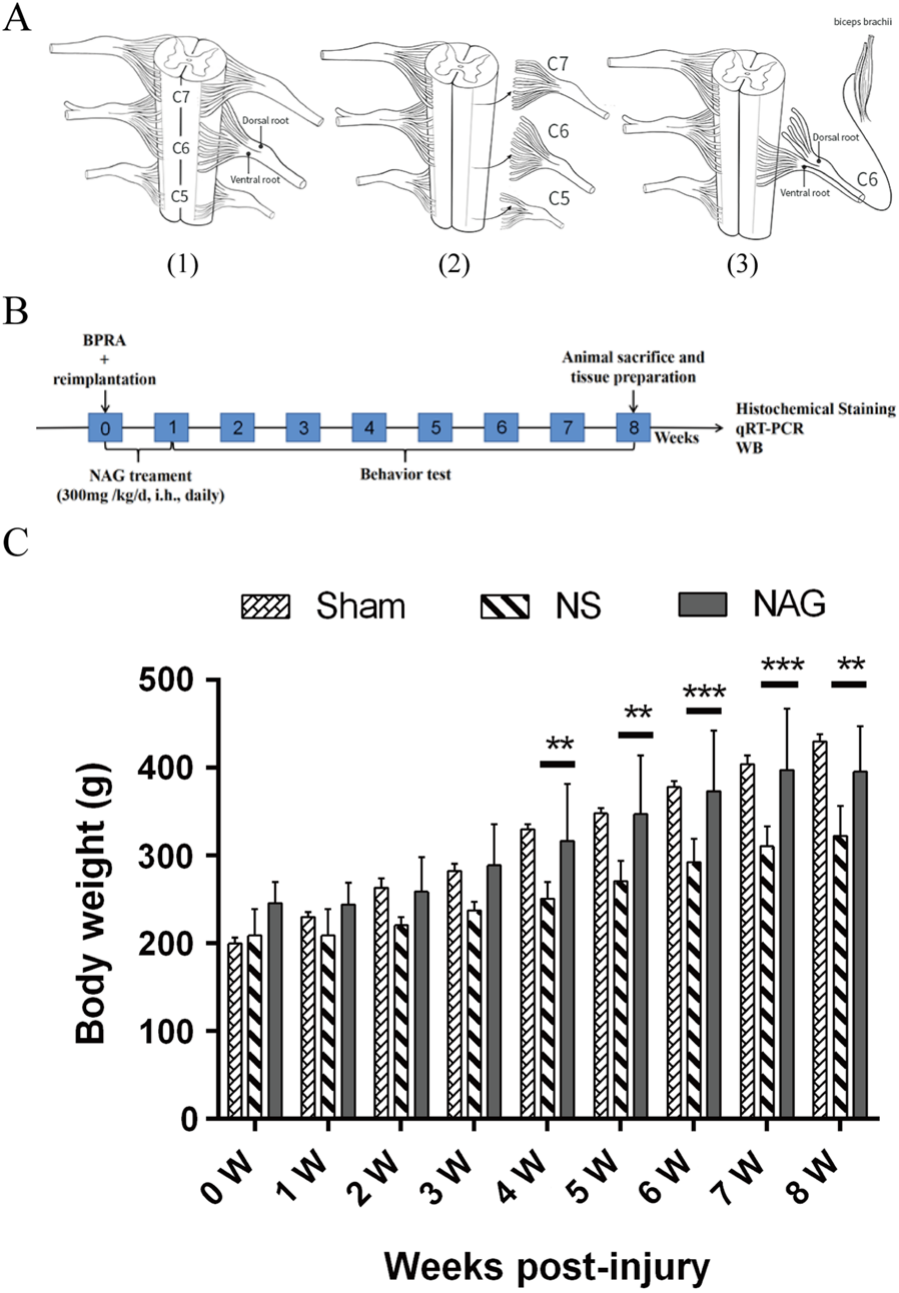
Brachial plexus root avulsion and reimplantation model, experimental design and rat body weight. **A** Schematic drawing of the surgical procedures. (1) Anatomical structure of normal C5–C7 spinal cord segments; (2) the right C5–7 ventral and dorsal roots were avulsed; and (3) the C6 ventral root was reimplanted to the surface of the corresponding spinal cord segment. **B** Schematic drawing of the workflow of the experimental design. **C** The average body weights in the subgroups at each time point are shown. The data shown are the mean ± SD in each group; ***P < 0.001, **P < 0.01, *P < 0.05. *NS* normal saline, *NAG* acetylglutamine

As shown in Fig. 1A, the surgical procedures were conducted as previously described [9, 15]. The rats were anesthetized by 5 µL/g 10% chloral hydrate via intraperitoneal injection. After the back hair was removed, the rats were fixed on a foam table in the prone position. After the skin, muscle, and fascia were cut, the C5, C6, and C7 spinal cord segments were accurately identified under a dissecting microscope. The right lamina segments were removed from the fourth cervical (C4) to the sixth cervical (C6) segment, and the C5–C7 segments of the spinal cord were exposed under a surgical microscope. After the dura mater was opened, the right-sided dorsal and ventral root C5–C7 segments were avulsed by being retracted with a fine glass hook. Then, the ventral root C6 segment was immediately reimplanted to the exact detached point after avulsion. To avoid any regeneration, the distal parts and proximal residual rootlets of the C5 and C7 segments of the spinal cord were cut and removed. In the Sham group, the C5–C7 segments of the spinal cord were exposed, and the dura mater was opened, but avulsion of C5–C7 dorsal and ventral roots was not performed. Care was taken to avoid any injury to the spinal cord. Finally, the muscles and skin were sutured, and the rats were placed back into their cages.

### Groupings and treatments

After the rat model of BPRA was established, the animals were randomly allocated into 3 groups, with 10 rats in each group: Sham group, NS group and NAG group. Rats in the NAG group were given an acetylglutamine solution (300 mg/kg d) via subcutaneous injection near the injury site, while rats in the Sham and NS groups were subcutaneously injected with an equal volume of normal saline for 7 consecutive days.

### Behavioral tests

#### Terzis grooming test

The Terzis grooming test was performed to determine motor function recovery of the affected forelimb as previously described [3, 9, 16]. Water was sprayed on the head of the rat from multiple directions to elicit a grooming reaction, and the rat was placed in a glass cylinder. After 1 − 3 mL of purified water was sprayed on the rat’s nose, the time to clear the water by forepaw movement was recorded by video for more at least 5 min by 2 researchers who were blinded to the groups. The scoring criteria were as follows: 0, the affected limb did not move at all; 1, the affected limb had the elbow flexion reflex but could not touch the nose; 2, the affected limb had the elbow flexion reflex and could touch the nose; 3, the affected limb had the elbow flexion reflex and could touch below the eyes; 4 points, the affected limb had the elbow flexion reflex and could touch the eyes; and 5 points, the affected limb had the elbow flexion reflex and could touch the ear and the part behind the ear. The highest score was recorded within 5 min according to this scoring criteria.

#### Cylinder test

A cylinder test was performed to evaluate the motor function recovery of the forelimbs as previously described [17, 18]. The rat was placed in a glass cylinder (50 cm in diameter, 70 cm in height). The number of times the right forepaw touched the cylinder wall was recorded by video until the left forepaw touched the cylinder 20 times.

#### Food pellet-taking test

The food pellet-taking test was conducted to evaluate the motor function of the affected forelimbs as described previously [17, 19]. After the rats were fasted for 24 h, they were videotaped as they ate uniform-sized cereals (spheres). The rats were then scored on the Irvine, Beatties and Bresnahan forelimb scale (IBB) scale for object support, grasping technique, finger movement, and joint position on a scale of 0–9.

#### Mechanical withdrawal threshold

Mechanical pain threshold testing was performed to determine changes in neuropathic pain behavior as previously described [20, 21]. After the rats were kept quiet, mechanosensitivity was tested with a range of Von Frey filaments (from thin to thick). The skin on the lateral plantar part of the foot was stimulated vertically with the fibrils, and the fibrils were slightly bent for 5 s. When the rat showed the paw withdrawal response, the same filament was selected for each interval of 5 min, the paw withdrawal response occurred more than 3 times in 5 consecutive measurements, and the corresponding fiber filament grams were recorded. If no paw withdrawal response was observed within the 5 s test period, adjacent fiber filaments were used until the paw withdrawal response occurred within 5 s.

### Tissue preparation

At 8 weeks after the surgery, the rats were sacrificed by chloral hydrate anesthesia, the tissues were collected for further analysis.

For qRT‒PCR analysis, total RNA was extracted from myocutaneous nerve tissue with TRIzol reagent (Invitrogen) according to the manufacturer’s protocol as previously described.

For Western blot analysis, spinal cord anterior horn tissue from the C5‒C7 segments was dissolved in RIPA buffer containing 1% PMSF in a volume of 100 µl (0754, Amresco). After homogenization with an electric tissue homogenizer (Fluka), the supernatant was collected after centrifugation at 4 °C for 20 min at 13,000 *g*.

For histological staining, the rats were transcardially perfused with saline followed by 4% paraformaldehyde (PFA) until they were rigid. Spinal cords, musculocutaneous nerves, and biceps were dissected and collected for further analysis. After being fixed in 4% PFA at 4 °C for 24 h, the tissues were transferred into PBS supplemented with 15% and 30% sucrose at 4 °C for 24 h. Then, the tissues were cut into sections using a sliding microtome (Leica CM1950, Leica).

### Histochemical staining

#### Nissl staining

Spinal cord sections were stained with Nissl stain for 2–5 min, and 0.1% glacial acetic acid was used to process the sections until the background was colorless or light blue and the Nissl bodies were dark blue.

#### Luxol fast blue (LFB) staining

Myelin stain A was preheated at 65 °C for thirty minutes. Musculocutaneous nerve sections were stained in myelin A for 4 h at 65 °C. The sections were processed in Myelin Stain B for 5 s and placed in Myelin Stain C for 10 s.

#### Hematoxylin and eosin (H&E) staining

Sections of the biceps brachii were first stained with hematoxylin for 3–5 min for nuclear staining. After being soaked in hematoxylin solution for 2–5 s, the sections were then stained with eosin staining solution for 5 min to ensure cytoplasmic staining. Finally, the tissue was dehydrated three times with absolute ethanol for 5 min each.

All images from Nissl staining, LFB staining and H&E staining were taken by light microscopy (MBF Nikon Microscope). The number of motor neurons, the diameters of nerves, the number of nerve fibers, the diameters of muscle fibers and the number of fibroblast nuclei were calculated using ImageJ 5.0 software.

### Quantitative real-time PCR (qRT‒PCR)

Total RNA was extracted from the C5–C7 segments of spinal cord tissues using TRNzol pure reagents (Tiangen Biotech, Beijing, China) and then electrophoresed on a 1% agarose gel to examine the purity. RNA concentration was determined by a NanoDrop 2000 instrument (Thermo Scientific, Rockford, IL, USA). One milligram of total RNA was reverse-transcribed to first-strand cDNA by using the PrimeScript™ RT Reagent Kit with gDNA Eraser (TaKaRa). The resultant cDNA was used as a template for subsequent PCR amplification with SYBR GREEN Master Mix (Solarbio, Beijing, China), and PCR was performed on an ExicyclerTM 96 Real-Time PCR System (ABI7500, Applied Biosystems). The qRT‒PCR primer sequences are listed in Table 1. A melting curve analysis was conducted to verify a single PCR product. The gene level was determined using the 2 ^− △△ CT^ method.

**Table 1:** Primer sequences for qRT-PCR

Genes		Toward (5'to 3')	Sequences(bp)
hmgcr	F	GTGGCCTCCATTGAGATCCG	258
hmgcr	R	ATGCACCGGGTTATCGTGAG	
prx	F	GAACTCTGGAGGTGTCTGGAG	88
prx	R	TTGAGGTCTTGCTGCCTGAG	
mpz	F	AGATGCCATTTCAATCTTCCAC	151
mpz	R	GTGCCGTTGTCACTGTAGTCT	
pmp22	F	CCTACTGCCCCCTTGCTTTG	101
pmp22	R	TAGCCTCAGGCACAAACTCG	
egr2	F	TCAGTCCAACCCCTCTCCAA	86
egr2	R	CATTTGCTCCTCGCACAACC	
L1CAM	F	CGAGTACAGGTCCCTGGAGA	163
L1CAM	R	TTGGCCGATGAAAGAGCCAT	
GAP-43	F	GATGCGGCCCCTTCAGAGGAA	134
GAP-43	R	GGCACATCGGCTTGTTTAGGC	
pou3f1	F	TGGGCCTAGCGCACCCTCAAT	154
pou3f1	R	ACCAAGCGGGCGTGGAAACCT	
ngfr	F	CATCTTGGCTGCTGTGGTCGT	197
ngfr	R	TCTGCGTATGGGTCTGCTGGT	
notch1	F	ACTATGGTTTGTGCAAGGATG	134
notch1	R	CATAAGCAGAGGTAGTAGTTGTCA	
sox2	F	CTCCATGACCAGCTCGCAGAC	165
sox2	R	GCCCTGGAGTGGGAGGAAGAG	
GAPDH	F	CGTATCGGACGCCTGGTT	83
GAPDH	R	AGGTCAATGAAGGGGTCGTT

**Table 2:** Primary antibody for Western blot

Anti-nNOS antibody	ab76067	Abcam
Anti-p-Akt1 antibody	ab8805	Abcam
Anti-GAP-43 antibody	ab75810	Abcam
Anti-p-p65 antibody	ab31624	Abcam
Anti-TNF-α antibody	ab215188	Abcam
Anti-IL-6 antibody	ab233706	Abcam
Anti-IL-1β antibody	ab254360	Abcam
Anti-IL-18 antibody	ab243091	Abcam
Anti-c-Fos antibody	ab222699	Abcam
Anti-NGF antibody	ab52918	Abcam
Anti-CGRP antibody	ab272713	Abcam
Anti-GSDMD antibody	ab219800	Abcam
Anti-NLRP3 antibody	ab263899	Abcam
Anti-Caspase-1 antibody	ab207802	Abcam
GAPDH	YM3029	Immunoway

### Western blot analysis

Twenty micrograms of protein were loaded onto sodium dodecyl sulfate‒polyacrylamide gel electrophoresis (SDS‒PAGE) gels at various concentrations for electrophoresis and transferred to a 0.45 μm nitrocellulose filter (NC) membrane (EMD Millipore Corporation, USA). The membranes were then blocked with 5% skimmed milk in TBST (Tris-buffered saline, 0.1% Tween 20) at RT for 1 h and incubated with the primary antibodies listed in Table 2 at 4 °C overnight. Subsequently, the membranes were washed 5 times with TBST and incubated with goat anti-mouse IgG horseradish peroxidase (HRP)-conjugated secondary antibodies (1:10,000, Tianderui Biotech., Beijing, China) for 40 min at RT. Immunoreactive proteins were detected by an enhanced chemiluminescence (ECL) kit (EMD Millipore Corporation, USA). Each protein band was quantified by Total Lab Quant V11.5 (Newcastle upon Tyne, UK).

### Statistical analysis

The data are expressed as the mean ± SD. Statistical analyses were performed by GraphPad Prism 6 software using Student’s t test. A *P* value < 0.05 was statistically significant.

## Results

### NAG treatment increased rat body weight following BPRA

To investigate the effect of NAG on the growth of rats subjected to BPRA, body weights were measured every week after injury. As shown in Fig. 1C, at 4 to 8 weeks after surgery, significant body weight loss was observed in the normal saline (NS) group compared to the sham group, while the body weights of NAG-treated rats were significantly increased.

### NAG treatment accelerated the recovery of motor function in the injured forelimbs of rats following BPRA

To evaluate the recovery of motor function in the injured forelimbs of rats after BPRA, grooming tests and cylinder tests were performed weekly, and the Food Pellet-taking Test was performed at 8 weeks postoperatively.

**Fig. 2:**
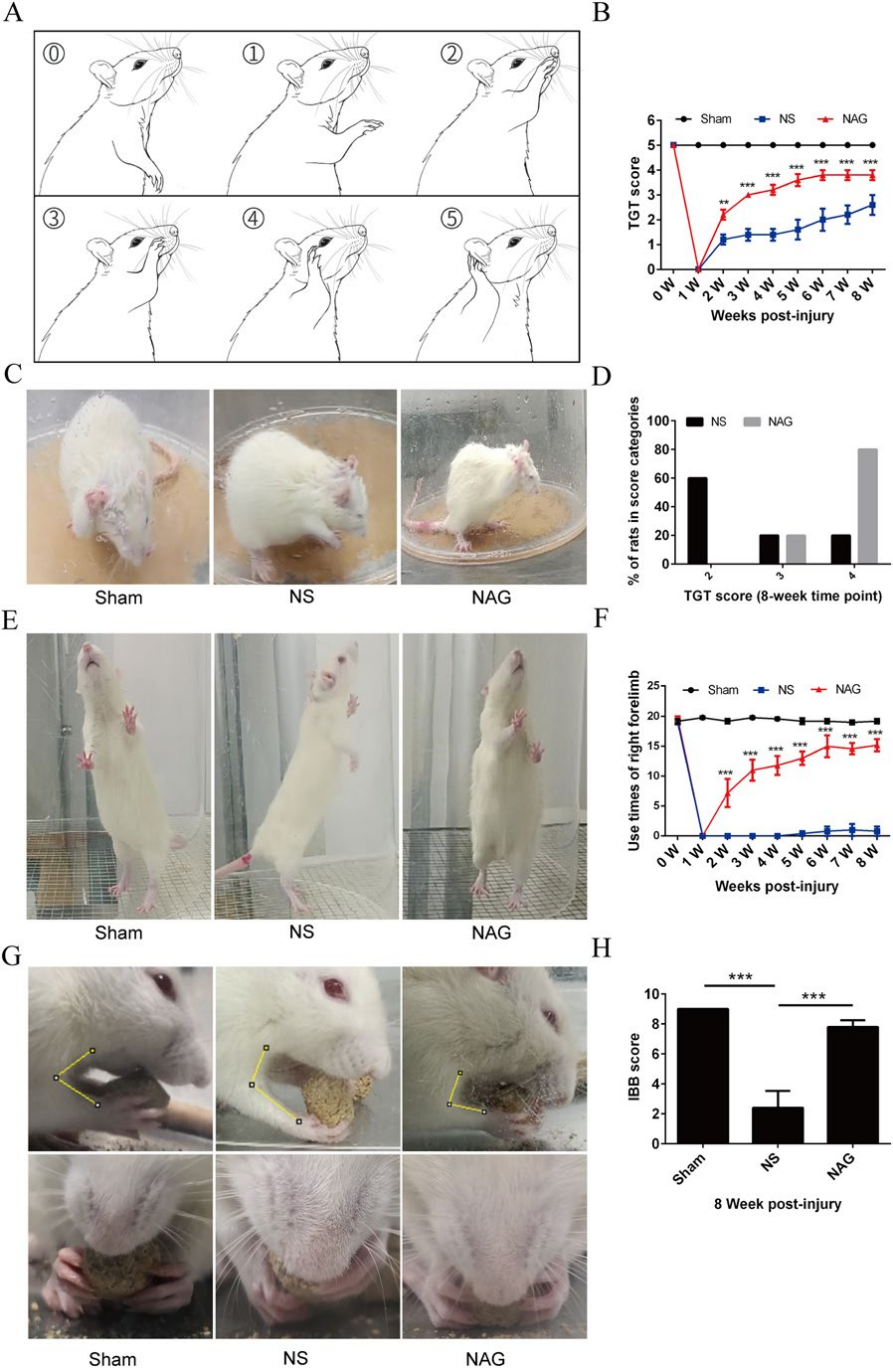
Effects of NAG treatment on motor functional recovery of the injured forelimbs. **A** Schematic drawing of the Terzis grooming test (TGT) and rating criteria. **B** Photographs of the TGT in each subgroup of rats. **C** Average TGT scores in each subgroup of rats at each time point. **D** The proportions of rats in each TGT score category in the NS and NAG groups at 8 weeks postsurgery. **E** Photographs of the cylinder test in each subgroup of rats. **F** Averaged use times of the right forelimb in each subgroup of rats at each time point. **G** Photographs of the Food Pellet-taking Test in each subgroup of rats. **H** Averaged IBB scores in each subgroup of rats at 8 weeks postsurgery. The data shown are the mean ± SD in each group; ***P < 0.001, **P < 0.01, *P < 0.05. *NS* normal saline, *NAG* acetylglutamine

Before surgery, all rats exhibited normal elbow flexion with a mean score of 5. As shown in Fig. 2A–D, rats in the NS and NAG groups had a TGT score of 0 one week after BPRA injury, indicating the complete loss of motor function and verifying the surgery’s success. Recovery of motor function was observed two weeks after surgery. Moreover, the average TGT scores 2 to 8 weeks after surgery in the NAG-treated group were significantly improved compared with those in the NS group. At 8 weeks postsurgery, a score of 4 was observed in 80% and 20% of rats in the NAG-treated and NS groups, respectively.

Before surgery, all rats exhibited similar abilities to use both forelimbs. As shown in Fig. 2E, F, compared to rats in the sham group, rats in the NS group exhibited decreased right forelimb use on the lesioned side (ipsilesional) after surgery. At 2 to 8 weeks postsurgery, right forelimb use in the NAG-treated group was significantly higher than that in the NS group.

To assess skilled forelimb function, we examined the ability to bring morsels to the mouth and lift and grasp small pellets with the fingers. To quantify the results, the IBB score was used. As shown in Fig. 2G, H, before surgery, all animals consumed food pellets with a mean score of 9. Compared to those in the sham group, the average IBB scores after surgery were decreased in the NS group. In contrast, the average IBB scores were increased in the NAG-treated group compared to the NS group.

### NAG treatment enhanced motoneuronal survival in the anterior horn of the spinal cord in rats following BPRA

To investigate the effect of NAG on motoneuronal survival after ventral root avulsion/reimplantation, Nissl staining was performed on spinal cord tissue. Moreover, we examined the expression levels of neuronal survival-associated proteins, including growth-associated protein-43 (GAP-43), phosphorylated (p)-Akt, and neuronal nitric oxide synthase (nNOS), by Western blot analysis.

**Fig. 3:**
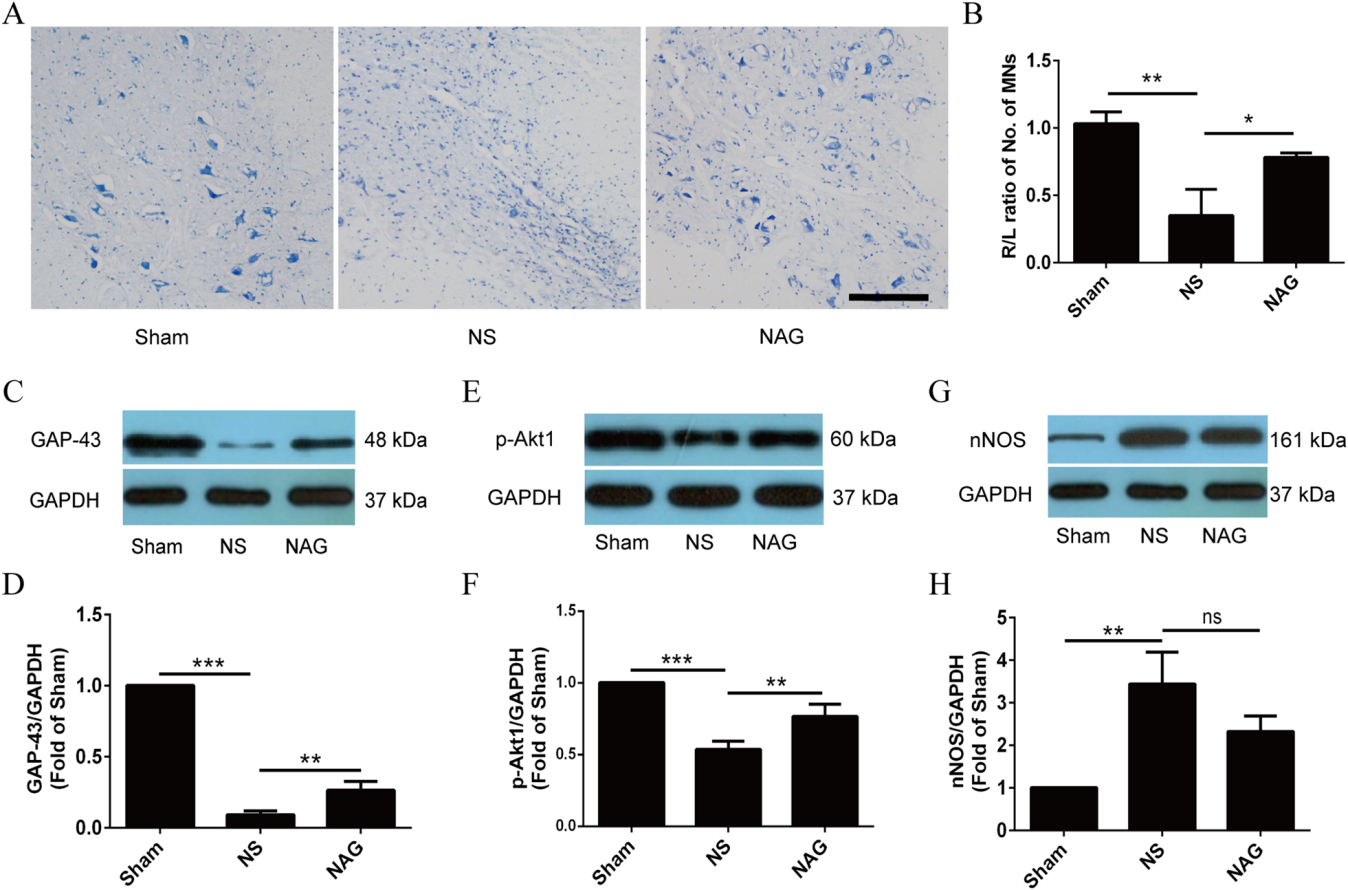
Effects of NAG treatment on histological alterations and motoneuronal survival in the spinal cord. **A** Histological images of Nissl-stained spinal cord sections in the groups. Scale bar represents 20 μm. **B** The survival rate of motoneurons was estimated as the percentage of the left (intact)/right (injury) motoneurons located in the ventral horn. The expression levels of GAP-43 (**C**,** D**), p-Akt1 (**E**, **F**) and nNOS (**G**,** H**) in the ventral horn of the spinal cord were determined using Western blotting. The data shown are the mean ± SD in each group; ***P < 0.001, **P < 0.01, *P < 0.05. *NS* normal saline, *NAG* acetylglutamine

As shown in Fig. 3A, B, avulsion of the root led to extensive motoneuron loss in the NS group compared to the Sham group. Moreover, treatment with NAG significantly increased motoneuron survival in the NAG group compared to the NS group.

As shown in Fig. 3G, H, the expression of GAP-43 and p-Akt in saline-treated spinal cords was significantly downregulated in comparison to that in the sham group, while nNOS levels were upregulated. Conversely, treatment with NAG significantly increased GAP-43 and p-Akt levels compared with those in saline-treated rats and downregulated nNOS levels.

### NAG treatment inhibited the expression of proinflammatory cytokines and the pyroptosis pathway in rats following BPRA

To investigate the underlying mechanism of the beneficial effect of NAG on the survival of injured motoneurons, the expression levels of several proinflammatory cytokines and pyroptosis pathway-related proteins in the spinal cord anterior horn were quantified by Western blotting.

**Fig. 4:**
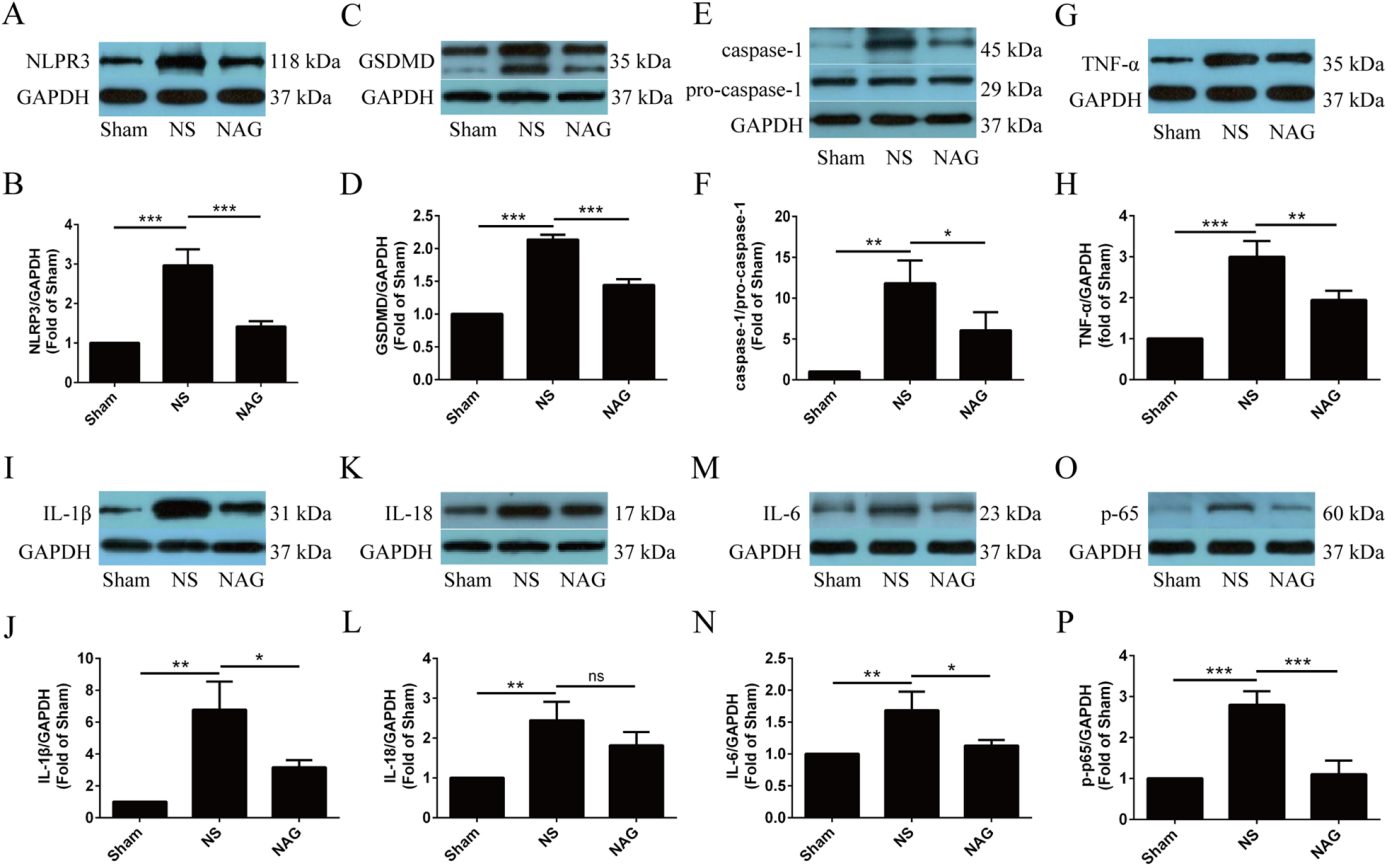
Effects of NAG treatment on the expression of IL-1β, IL-18, IL-6, TNF-α, p-p65, NLRP3, GSDMD, pro-caspase-1 and caspase-1 in the ventral horn. The expression levels of NLRP3 (**A**,** B**), GSDMD (**C**,** D**), caspase-1/pro-caspase-1 (**E**,** F**), TNF-α (**G**,** H**), IL-1β (**I**,** J**), IL-18 (**K**, **L**), IL-6 (**M**,** N**) and p-p65 (**O**,** P**) in the ventral horn of the spinal cord were determined using Western blotting. The data shown are the mean ± SD in each group; ***P < 0.001, **P < 0.01, *P < 0.05. *NS* normal saline, *NAG* acetylglutamine

As shown in Fig. 4A–P, after BPRA injury, the levels of IL-1β, IL-18, IL-6, TNF-α and p-p65 in saline-treated rats were significantly higher than those in the sham group, while IL-1β, IL-6, TNF-α and p-p65 protein expression was significantly downregulated in NAG-treated rats. Although IL-18 levels were decreased by NAG treatment, the difference was not significant. Moreover, NLRP3, GSDMD, and caspase-1 protein expression levels in the anterior horn of the spinal cord in the NS group were higher than those in the sham group. The protein expression of the NLRP3, GSDMD, and caspase-1 was downregulated in the NAG-treatment group.

### NAG treatment facilitated axonal remyelination in the myocutaneous nerves of rats following BPRA

To determine the effects of NAG on the morphology of myocutaneous nerves in BPRA rats, myocutaneous nerve tissue was stained with LFB. We further examined whether the improvement in myocutaneous nerve morphology was related to axonal remyelination of motoneurons. qRT-qPCR was performed to assess changes in the expression of myelination-related genes.

**Fig. 5:**
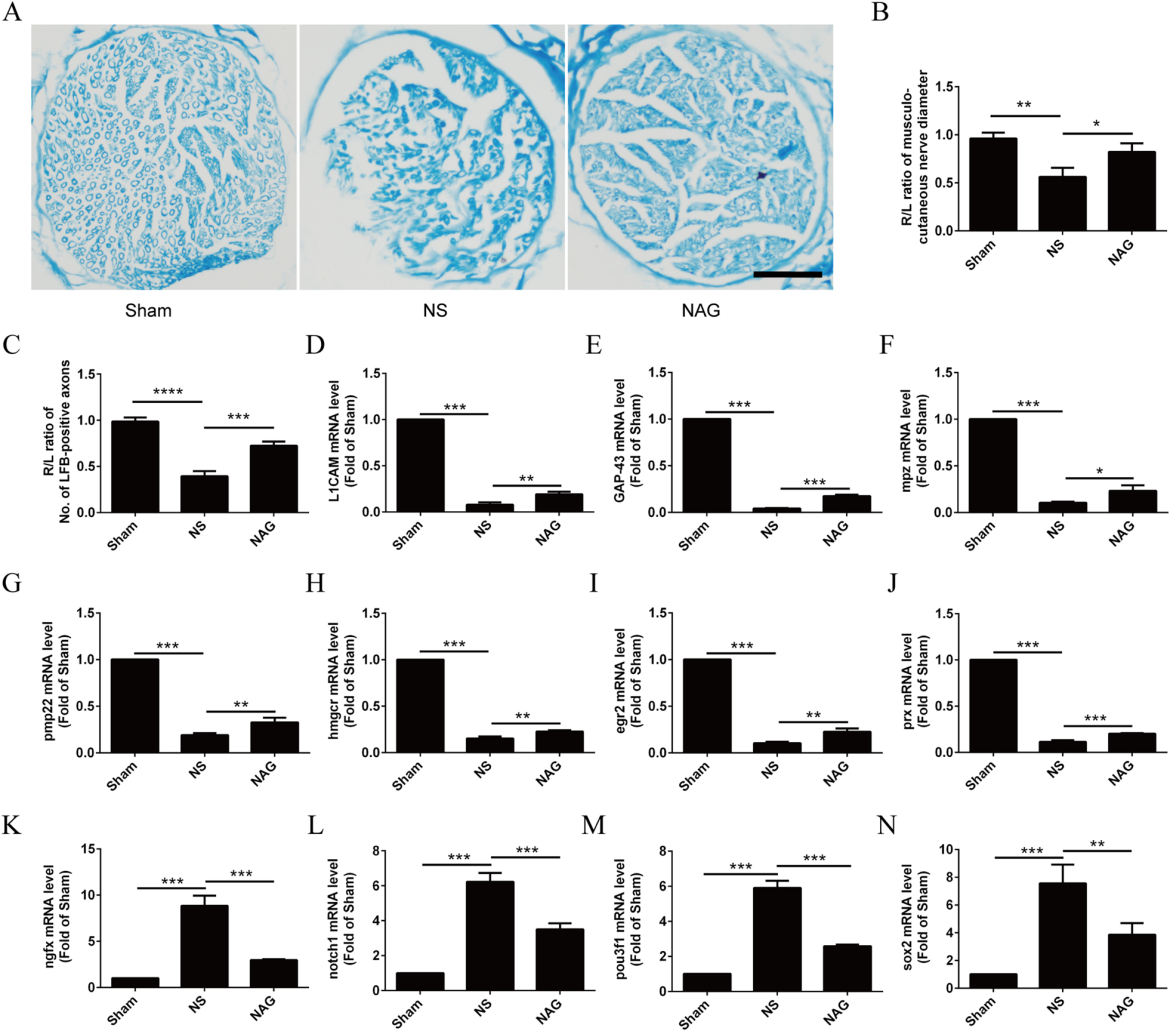
Effects of NAG treatment on histological alterations and the remyelination of musculocutaneous nerves. **A** Histological images of LFB-stained musculocutaneous nerve sections in the groups. Scale bar represents 20 μm. **B** Average ratio of the diameter of musculocutaneous nerves of the right (injury) to the left (intact) side at 8 weeks postsurgery. **C** Average ratio of the number of LFB-positive axons of the right (injury) to the left (intact) side at 8 weeks postsurgery. **D–J** The mRNA levels of myelination-associated genes (egr2, GAP-43, hmgcr, L1CAM, mpz, pmp22, and prx) were determined using qRT‒PCR. **K–N** The mRNA levels of demyelination-associated genes (ngfr, notch1, pou3f1, and sox2) were determined using qRT‒PCR. The data shown are the mean ± SD in each group; ***P < 0.001, **P < 0.01, *P < 0.05. *NS* normal saline, *NAG* acetylglutamine

As shown in Fig. 5A, B, a decline in the number of LFB-positive axons was observed in saline-treated rats in comparison to rat in the sham group. However, the number of LFB-positive axons on the lesioned side in the NAG group was higher than that in the NS group. Moreover, we observed that the diameter of the musculocutaneous nerve in the NS group was significantly smaller than that in the sham group. In addition, the diameter of the musculocutaneous nerve in the NAG-treated group was higher than that in the saline-treated group.

As shown in Fig. 5C–N, remyelination-associated genes (egr2, GAP-43, hmgcr, L1CAM, mpz, pmp22, and prx) were downregulated in response to injury compared to those in the sham group. However, these genes were upregulated in the NAG-treated group after injury. The opposite findings were observed for demyelination-associated genes (ngfr, notch1, pou3f1, and sox2).

### NAG treatment alleviated biceps brachii atrophy in rats following BPRA

To determine the degree of muscle atrophy after surgery, the biceps brachii muscles of the right (injury) and left (intact) forelimbs were weighed, and the right/left biceps brachii weight ratio was calculated. Then, histopathological changes in the biceps tissue were evaluated by hematoxylin and eosin (H&E) staining.

**Fig. 6:**
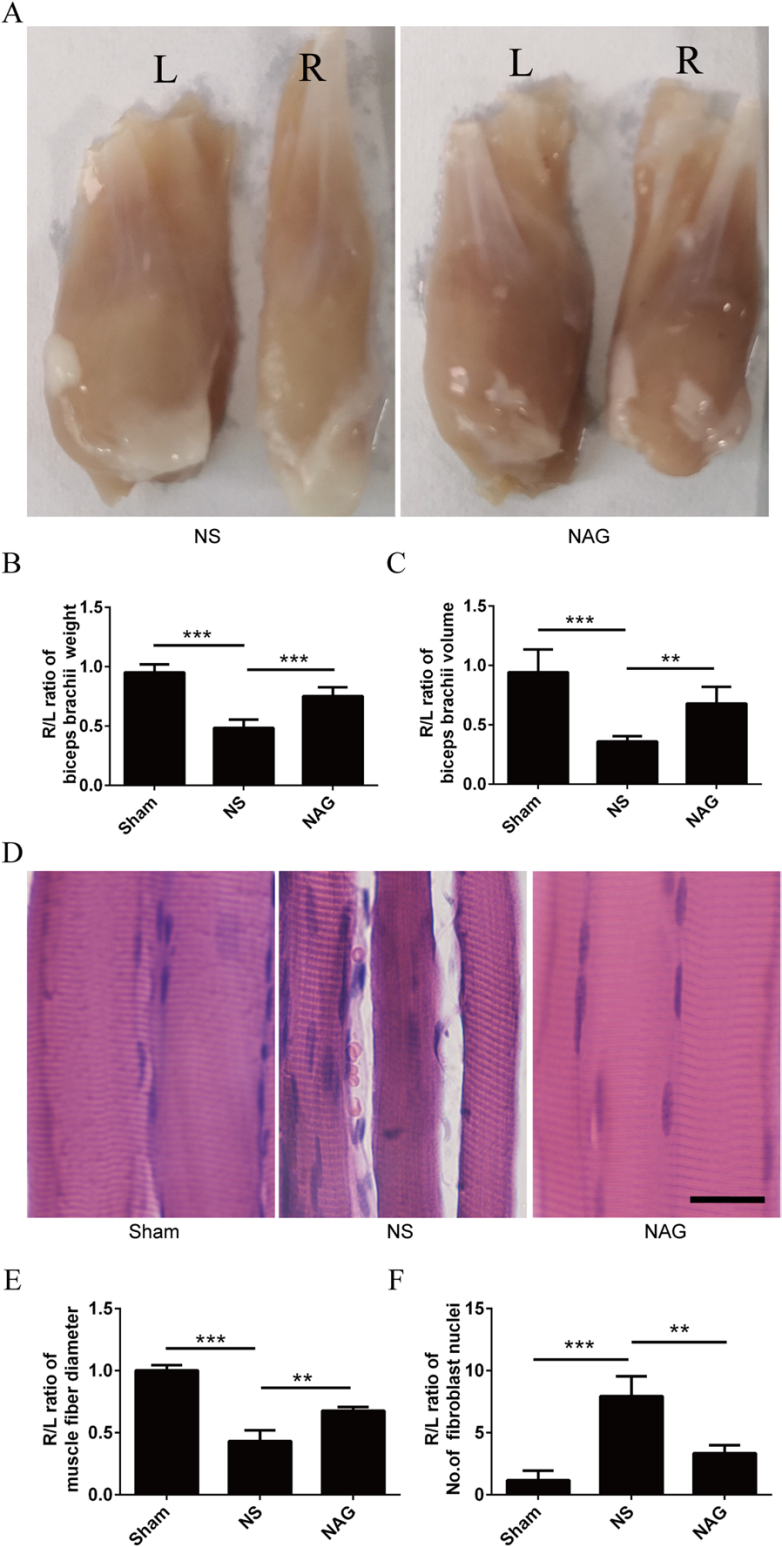
Effects of NAG treatment on the weight and histological alteration of biceps. **A** Representative photographs of biceps muscles from both ipsilateral and contralateral regions of the avulsed rats. Scale bar represents 2 mm. **B** Average ratio of biceps muscles wet weight of the right (injury) to the left (intact) side at 8 weeks postoperatively. **C** Average ratio of biceps muscle volume of the right (injury) to the left (intact) side at 8 weeks postoperatively. **D** Histological images of longitudinal bicep muscle sections with H&E staining from all groups. Scale bar represents 100 μm. **E** Average ratio of the muscle fiber diameter of the right (injury) to the left (intact) side. **F** The extent of fibrosis was calculated as the ratio of the number of fibroblast nuclei in the right (injury) to the left (intact) side. Data shown are mean ± SD in each group; ***P < 0.001, **P < 0.01, *P < 0.05. *NS* normal saline, *NAG* acetylglutamine

As shown in Fig. 6A–C, compared to that in the sham group, a significant decline in the weight of biceps brachii muscles on the lesioned side was observed in the NS group. However, the weight of the biceps brachii muscles on the lesioned side was significantly increased in the NAG-treated group compared to the NS group. Similar patterns were observed for biceps brachii muscle volume.

As shown in Fig. 6D–F, saline-treated muscle fibers exhibited significantly smaller diameters and higher amounts of fibroblasts in comparison to those in the sham group, showing clear signs of muscular atrophy. Conversely, NAG-treated muscle fibers were larger with clear myocyte nuclei and no apparent fibrosis and were morphologically similar to normal fibers.

### NAG treatment attenuated neuropathic pain in rats following BPRA

The mechanical withdrawal threshold (MWT) was determined weekly based on the withdrawal responses of the right forepaw to mechanical stimulation delivered by von Frey filaments. Moreover, we used Western blotting to quantify the neuropathic pain-related proteins calcitonin gene-related peptide (CGRP), nerve growth factor (NGF), and c-FOS in the spinal dorsal horn.

**Fig. 7:**
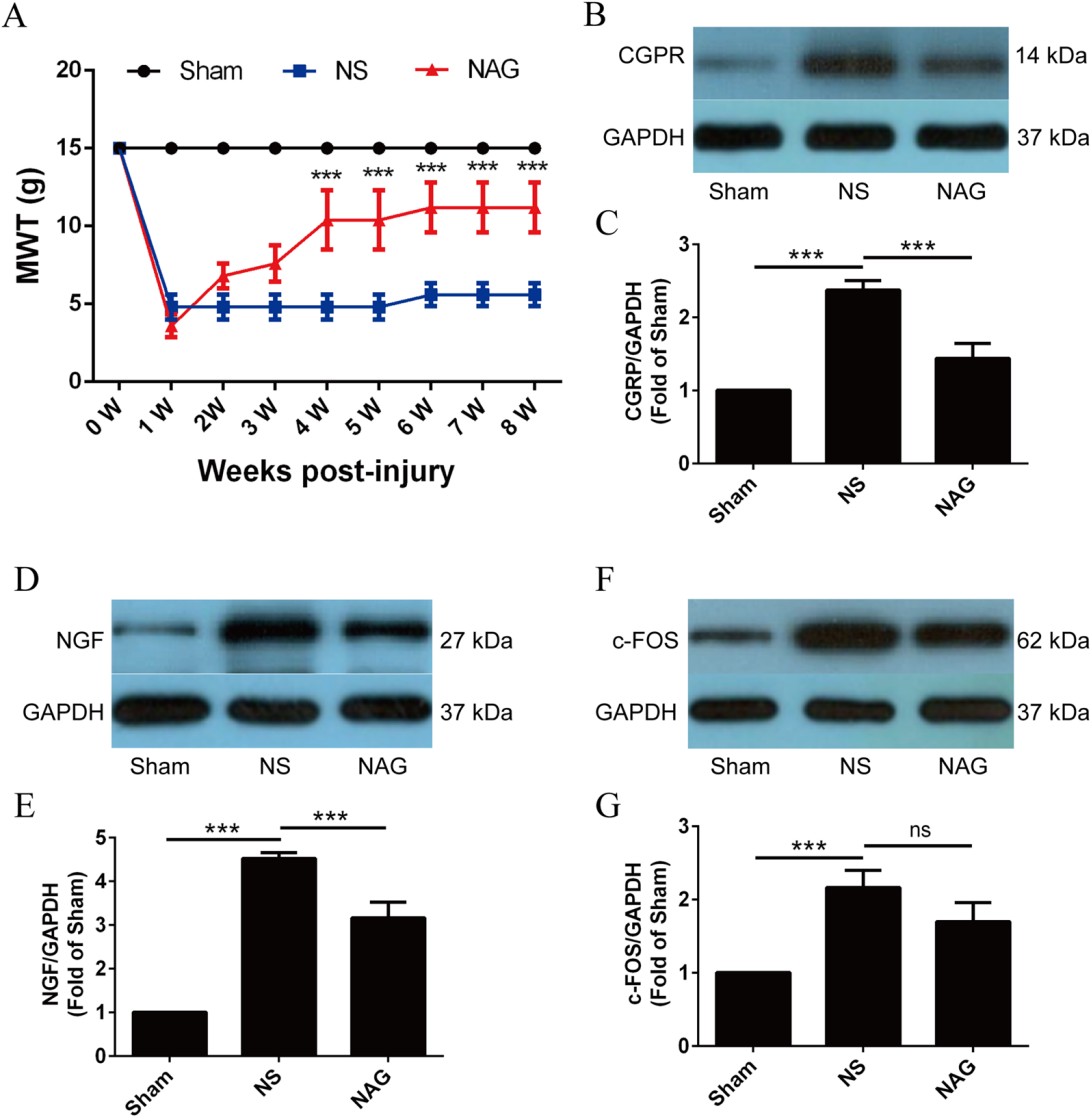
Effects of NAG treatment on changes of MWT and the expressions of CRGP, NGF and c-FOS in the dorsal horn. **A** Average MWT in each subgroup of rats at each time point. The expression levels of CRGP (**B**,** C**), NGF (**D**, **E**) and c-FOS (**F**, **G**) in the dorsal horn of the spinal cord were determined using WesternBlot. Data shown are mean ± SD in each group; ***P < 0.001, **P < 0.01, *P < 0.05. *NS* normal saline, *NAG* acetylglutamine

As shown in Fig. 7A, we used rats with a preoperative mechanical pain threshold of 15 g to compare changes in the MWT after BPRA injury. Compared with that in the sham group, the MWT of the right forepaw of saline-treated rats was significantly decreased at 4–8 weeks postsurgery. Notably, in rats that were treated with NAG, the MWT of the right forepaw was significantly higher than that in the NS group.

As shown in Fig. 7B–G, the protein levels of CGRP, NGF and c-FOS were increased in the NS group, but this change was reversed for CGRP and NGF after treatment with NAG, which also resulted in decreased protein levels of c-FOS.

## Discussion

In previous studies, we revealed the effects of artemisinin, berberine and neuregulin-1 on motor function recovery after BPRA [9, 15, 16]. In the present study, we demonstrated that NAG treatment combined with nerve reimplantation contributed to improved functional recovery after BPRA by increasing motoneuron survival, accelerating motor axonal remyelination, reducing muscle atrophy and improving sensory recovery, which may be associated with inhibition of the inflammatory response and the pyroptosis pathway.

Currently, neurological functions are commonly assessed to determine the degree of injury and the influence of potential treatment strategies [16]. Although exogenous neurotrophic factors, including glial cell-derived neurotrophic factor (CDNF) and brain-derived neurotrophic factor (BDNF), have been shown to reduce the loss of motoneurons after BPRA, their application at the clinical level to treat human cases of BPRA is largely limited by their short half-life and inability to cross the BBB [3]. In recent years, NAG has been used to treat various diseases, including hepatic coma, hemiplegia, brain trauma, and cerebral apoplexy sequelae, because of its anti-inflammatory and neuroprotective effects [22]. In this study, NAG-treated rats exhibited motor function recovery after BPRA, and there were higher mean TGT and IBB scores and increased use of affected forelimbs in the NAG-treated groups in comparison to the NS group.

Maintaining motoneuron survival after injury for functional restoration is necessary. It has been established that growth-associated protein-43 (GAP-43), phosphorylated (p)-Akt and neuronal nitric oxide synthase (nNOS) are important proteins involved in neuron survival and axon growth. GAP-43, a specific phosphoprotein on the vertebrate nerve cell membrane, is widely used as a marker of neuronal development, regeneration and synaptic plasticity [23]. Moreover, an increasing body of evidence suggests that suppressing nNOS can protect against neuronal death subject to peroxynitrite cytotoxicity [3, 15]. In addition, the key role of Akt phosphorylation in neuronal growth and survival in traumatic lesions is widely acknowledged [24, 25]. The present study showed that motoneuron survival was enhanced in the NAG-treated group, which had increased GAP-43 and p-Akt levels and reduced nNOS expression.

Spinal root avulsion is characterized by the excessive activation of lesion-infiltrating microglia/macrophages and astrocytes [7, 26] that produce proinflammatory cytokines to inhibit neuronal survival [27]. Pyroptosis is a well-recognized form of programmed cell death that involves proinflammatory activity. In this regard, when the body is subjected to intracellular and extracellular signaling pathways, noxious stimuli induce intracytoplasmic inflammasome formation via the caspase-4/5/11-dependent nonclassical pyroptosis pathway and/or caspase-1-dependent classical apoptotic pathway. This phenomenon leads to caspase-4/5/11 or caspase-1 activation, which upregulates the secretion of proinflammatory cytokines, including interleukin-18 (IL-18) and interleukin-1β (IL-1β), resulting in pyroptosis [28–30]. There is ample evidence to suggest that pyroptosis occurs extensively in the diseased central nervous system [31, 32]. Accordingly, regulating pyroptosis may regulate disease-associated inflammation. Our findings suggested that NAG could prevent motoneuron loss by downregulating the levels of proinflammatory cytokines, including TNF-α, IL-6-, IL-1β, IL-18, and p-p65. Overall, we found that NAG could prevent motoneuron loss by downregulating the levels of NLRP3, Caspase-1, and GSDMD after BPRA.

Importantly, the reimplanted ventral spinal root affords a permissive microenvironment for axonal elongation and regrowth in BPRA rats. However, reimplantation alone is not sufficient for reinnervation in humans and rodents [5]. Consistently, Han et al. reported that NAG could promote neurite outgrowth and axonal regeneration with thicker remyelinated axons [33]. Indeed, efficient remyelination of the regenerated axons in BPRA rats has been associated with early functional recovery [34]. Importantly, NAG treatment can accelerate the regeneration of axons that innervate target muscles. In our study, treatment with NAG significantly increased the diameters of the musculocutaneous nerve and the number of LFB-positive axons. This observation may be explained by enhanced axonal branching or accelerated axonal extension induced by NAG. Moreover, we showed that remyelination-associated genes (L1CAM, GAP-43, pmp22, mpz, hmgcr, egr2, and prx) were downregulated, while demyelination-associated genes (ngrx, notch1, pou3f1, and sox2) were upregulated in NAG-treated rats, indicating the ability of NAG to potentiate axonal remyelination.

It is widely acknowledged that motor neuron axons deliver neurotransmitters into the synaptic cleft and exert a trophic effect on the controlled muscle fibers. Interestingly, after denervation, morphological alterations such as shrunken sarcoplasm and fibrosis are induced in muscle fibers [35]. During muscle reinnervation, muscle fibers are clustered but are not scattered as they are in the uninjured biceps. Because of the death of MNs and failure to target myocytes, the muscle fibers are reinnervated by the nearest axon sprouts, causing fiber-type grouping [36]. In our study, we found that NAG could efficiently alleviate muscle atrophy in rats subjected to avulsion/reimplantation, which was associated increased biceps muscle weight and volume, larger fiber size and decreased number of fibroblast nuclei.

Neurogenic pain is a common and refractory complication after BPRA injury [37]. In addition to motor and sensory deficits, pain can be equally debilitating. BPRA pain has been characterized as rapid (an effect that occurs immediately after the trauma) and intermittent shooting pain, which may be observed at sites distant from the lesion. The pain states are generated and maintained by the activation of microglia and astrocytes in the spinal cord, which typically lasts 3 months after BPA [38]. Various nociceptive stimuli can activate c-Fos (a marker associated with pain) in the spinal cord [20]. NGF and CGRP have been shown to play critical roles in the molecular mechanisms of inflammatory-mediated disorders and are closely associated with nerve pain. Consistently, we observed that NAG could significantly reduce the MWT and protein levels of NGF and CGRP, suggesting that NAG alleviates pain.

Overall, this study is the first to provide compelling evidence that acetylglutamine promotes functional motor recovery by enhancing motoneuronal survival and axonal remyelination after brachial plexus root avulsion in rats. Moreover, acetylglutamine can inhibit pyroptosis and attenuate the inflammatory response in the anterior horn of the spinal cord, leading to improved motoneuron survival. Importantly, we demonstrated that acetylglutamine could reduce neuropathic pain following brachial plexus avulsion.

Although the results seem exciting, because promoting the survival of injured motor neurons is a prerequisite for functional motor recovery after BPRA, so, in our present study, we mainly concentrated on promoting the motoneuron survival to facilitate motor recovery. Further studies related to the effect of NAG on regeneration of motoneuron after BPRA are no doubt needed to be performed. Overall, acetylglutamine has robust prospects for clinical applications to treat brachial plexus avulsion.

## Data Availability

All data generated or analyzed during this study are included in this published article.
